# Annual Research Review: Anterior Modifiers in the Emergence of Neurodevelopmental Disorders (AMEND)—a systems neuroscience approach to common developmental disorders

**DOI:** 10.1111/jcpp.13372

**Published:** 2021-01-11

**Authors:** Mark H. Johnson, Tony Charman, Andrew Pickles, Emily J. H. Jones

**Affiliations:** ^1^ Centre for Brain and Cognitive Development Department of Psychological Sciences Birkbeck, University of London London UK; ^2^ Department of Psychology University of Cambridge Cambridge UK; ^3^ Department of Psychology Institute of Psychiatry, Psychology and Neuroscience King’s College London London UK; ^4^ Department of Biostatistics and Health Informatics Institute of Psychiatry, Psychology and Neuroscience King’s College London London UK

**Keywords:** Neurodevelopmental disorders, autism spectrum disorders, brain development

## Abstract

We present the Anterior Modifiers in the Emergence of Neurodevelopmental Disorders (AMEND) framework, designed to reframe the field of prospective studies of neurodevelopmental disorders. In AMEND we propose conceptual, statistical and methodological approaches to separating markers of early‐stage perturbations from later developmental modifiers. We describe the evidence for, and features of, these interacting components before outlining analytical approaches to studying how different profiles of early perturbations and later modifiers interact to produce phenotypic outcomes. We suggest this approach could both advance our theoretical understanding and clinical approach to the emergence of developmental psychopathology in early childhood.

## Introduction

Neurodevelopmental disorders such as autism spectrum disorder (ASD) and attention deficit/hyperactivity disorder (ADHD) can only be fully understood by taking a prospective longitudinal approach from infancy. Such programmes have now come of age, with several groups having now collected data on cohorts for over a decade (for reviews, see Johnson, Gliga, Jones, & Charman, 2015; Jones, Gliga, Bedford, Charman, & Johnson, 2014; Szatmari et al., 2016; Varcin & Nelson, 2016). The prospective longitudinal design of infants with a family history of ASD has now also been extended to ADHD, anxiety and other common or co‐occurring traits (Cantiani et al., 2019; Johnson, Gliga, et al., 2015; Miller et al., 2020). In this review, we present a new framework (Anterior Modifiers in the Emergence of Neurodevelopmental Disorders, AMEND) designed to enable prospective studies to fulfil their potential to address major challenges in the field of neurodevelopmental disorders. These challenges include: (a) the lack of a simple linear causal pathway in the emergence of atypical phenotypes; (b) the need to accommodate both the dimensional and categorical nature of neurodevelopmental disorders; (c) the need to identify the appropriate levels for mechanistic explanations; and (d) the need to understand the factors that can protect against deviations from typical developmental pathways, or modify a trajectory to ameliorate atypical symptoms.

With regard to the first challenge, prospective longitudinal designs in principle allow causal pathways to be delineated as they unfold. Decades of research have revealed that ASD and ADHD do not result from simple mechanistic causal pathways (Faraone et al., 2015; Lord et al., 2020). Developmental pathways to atypical phenotypes show both convergence (‘equifinality’) and divergence of trajectories (‘multifinality’; Cicchetti & Rogosch, 1996). With regard to convergence, it is well established that a large variety of different genetic, environmental and brain factors can all significantly increase the likelihood of a later ASD diagnosis (Lord et al., 2020). Conversely, seemingly identical causal factors can raise the likelihood of different atypical phenotypes in different individuals. For example, the genetic condition neurofibromatosis 1 increases the likelihood of both subsequent ASD and ADHD diagnoses (Garg et al., 2013; Vogel, Gutmann, & Morris, 2017). The existence of these convergence and divergence phenomena counsels against overly simplistic reductionist approaches to understanding these phenotypes, as the mapping to underlying genetic and molecular factors is likely probabilistic and partially stochastic (White, 2019). Instead, for an informative mechanistic explanation of these natural phenomena, we need to understand the emergent properties of the system as it changes over developmental time using a systems neuroscience approach (Ahn, Tewari, Poon, & Phillips, 2006). Systems neuroscience is a branch of systems biology, in which the aim is to understand emergent properties that arise from combinations of factors interacting as a whole system. This approach may be particularly valuable when studying developmental phenomena where events unfold over multiple time scales (Davies, 2017), such as those assessed within the context of prospective longitudinal studies. In a systems neuroscience approach, these principles are applied to the study of the brain from the molecular level to that of whole brain and cognitive systems.

Second, prospective longitudinal studies from infancy can illuminate the contexts in which developmental disorders are best characterised as punctate categories (i.e. categorical diagnosis), or as graded zones of a multidimensional space (dimensional trait measurements). Pickles & Angold (2003) discuss this issue from a statistical perspective and conclude that it is useful to maintain both perspectives depending on the context and nature of the question under investigation, with an analogy being drawn to light being studied as either a wave or a particle. From a developmental systems neuroscience perspective, phenotypic outcomes can be considered as being basins of attraction in a multidimensional state space in which individual variation can emerge both directly from genetic variation and indirectly from the nonlinear developmental process (Davies, 2017; Davila‐Velderraine & Alvarez‐Buylla, 2014; Huang, Eichler, Bar‐Yam, & Ingber, 2005). Prospective longitudinal studies have the potential to help us integrate both categorical and dimensional frameworks because they involve the population of interest being defined independently of, and prior to, clinical diagnosis.

A third issue central to understanding atypical development is selecting the most appropriate levels of investigation and explanation for the behavioural phenomena observed. Reductionist approaches are very common in biomedicine, in which it is assumed that a single underlying genetic, neurochemical, cognitive or behavioural component will be sufficient to explain and treat a given condition. However, experience with complex multifactorial physical (e.g. diabetes) and mental health conditions has shown this approach to be inadequate as ‘there are circumstances in which the complex interplay between parts yields a behaviour that cannot be predicted by the investigation of the parts alone’ and ‘reductionism is less helpful for systems where interactions between components dominate the components themselves in shaping the system‐wide behaviour’ (Davila‐Velderraine & Alvarez‐Buylla, 2014). Taking a modern systems neuroscience approach to biomedical issues requires a synthetic approach in which we focus on how component–component interactions results in emergent phenotypes, both common and rare (Johnson, 2013, 2015). This necessitates the study of development at multiple levels from genetic to cellular, and from brain systems to behaviour, and drives the need for developing a framework for explaining different phenotypic outcomes in terms of the systems behaviour of interacting components over developmental time.

The final challenge that can be addressed through prospective studies from infancy is the identification of resilience and protective factors. In child psychiatry, the concept of resilience is commonly used to refer to the extent to which an individual withstands or recovers from early disturbance of their developmental trajectory to achieve a typical outcome, or at least the avoidance of significant psychopathology (Cicchetti, 2013; Cicchetti & Curtis, 2007; Masten, 2007). Protective factors are genetic or environmental factors that act simultaneously or in parallel with risk factors to mitigate their effects, or later in trajectories to promote resilience. Traditionally studied examples of protective factors are female sex, intellectual functioning, maternal education, social support and family climate, and breastfeeding (Crush, Arseneault, Jaffee, Danese, & Fisher, 2018; Walker et al., 2011; Wüstner et al., 2019). Only recently have investigators turned to study specific brain systems that may act to increase resilience through their ability to compensate for, or reduce atypicality in, earlier developing brain networks. Protective, resilience and risk factors can be best disentangled in a prospective design because of their distinct profiles of interaction over developmental time (see Table 1 for a definition of our use of terms). Of note, rigorous identification of protective and predisposing factors requires assessment of causality. Within the field of epidemiology, a collapse of confidence in the ability to provide causal attributions that could be replicated in trials has led to demands for much more rigorous testing of claims as to causation. More formal methods of analysis can help, notably those based on a detailed understanding of concepts such as counterfactuals and directed acyclic graphs that have yet to be widely adopted in the developmental literature. Though perhaps not always essential (Krieger & Davey Smith, 2016), the need to understand and make clear the weak points of any inferential analysis is well accepted.

**Table 1:** Consideration of the relation between our framework and the concepts of risk and protective factors in psychiatry

Concept	Definition and relation to AMEND
Early‐stage Processing (ESP)	Markers that reflect the activity of brain systems engaged in sensory and motor functions in early development; without moderation by neurocognitive modifiers atypical early‐stage processing leads to atypical phenotypic outcomes; in this sense, atypical early‐stage processing can be a *risk factor* (see below).
Neurocognitive modifier system (NMS)	Trajectory‐modifying brain systems associated with anterior regions of cortex; they moderate the association between ESP and phenotypic outcome such that strong NMS activity pushes trajectories towards typical outcome; as these systems act in parallel and/or later than atypical ESP they constitute *resilience* (see below); NMS also act to influence typical developmental trajectories, resulting in some individual differences
Risk/Risk factor	‘Risk factors’ are typically defined as variables associated with an increased risk of later disease; in turn, ‘risk’ itself is often defined as the degree of probability of loss, injury, peril or hazard. Two elements of these definitions can be problematic in the context of developing a mechanistic understanding of neurodevelopmental disorders: (a) the term ‘risk’ implies relation to a negative outcome, and as such implies an inappropriate value judgement about autism and ADHD; (b) risk factors are defined by their probabilistic relation to a particular outcome, which while useful in a clinical context, is less helpful for mechanistic accounts of developmental trajectories. In AMEND, atypical ESP factors at a given age statistically associate with later phenotypic status and thus represent risk markers. However, this risk is reduced by the degree of action of NMS.
Resilience/ Protective factors	*Protective factors* are typically defined as contexts or attributes that mitigate or eliminate risk at its outset. *Resilience* refers to mechanisms that act concurrently with, or later than, initial risk factors to restore typical phenotypic outcomes. NMS underlie resilience, but initial individual genetic variants associated with strong NMS can be regarded as protective factors.

Given the potential of prospective designs to critically inform models of neurodevelopmental disorders, what have we learned so far? The initial conceptual framework underlying the first wave of prospective ASD family studies was to identify markers or precursors that were different in infants with later categorically defined ASD relative to infants with other outcomes. Further, the utility of particular brain, cognitive or behavioural markers has sometimes been judged in terms of the sensitivity and specificity with which they predict later clinical diagnosis (Hazlett et al., 2017); however, significant group differences in infant markers between those with and without later ASD diagnosis have typically not performed well for prediction at the individual level. To date, a broad overview of the literature on early markers for autism (the best studied condition to date) indicates that the sensitivity and specificity of markers for ASD increases over the first two years of life and is highest for markers closest to the level at which ASD symptoms are measured (i.e. in behaviour; Bussu, Jones, Charman, Johnson, & Buitelaar, 2018; Chawarska et al., 2014; Ozonoff et al., 2015). In contrast, in the first six months, many atypicalities appear sensorimotor in nature and few differences in behavioural measures of social communication are seen (Jones et al., 2014); however, from 10 to 24 months, ASD‐related behavioural symptoms gradually emerge (e.g. Ozonoff et al., 2010). Anatomical and neurofunctional associates of later‐emerging ASD implicate widespread alterations across regions (Hazlett et al., 2017) and processing stages (Tye, Bussu et al. in press) and do not highlight differences that are discrete and localisable to particular functions. Taken together, this profile further supports the need to understand atypical behavioural phenotypes in terms of the interaction between large‐scale brain networks (i.e. a systems neuroscience approach).

A simple explanation of the observation that some infant predictors of later‐emerging ASD lack sensitivity and specificity on an individual level is that measurement precision is poor in data from young infants, blunting the potential for association with later outcome. Many factors do complicate data acquisition from early infancy, including short attention spans, high levels of motion, and a limited behavioural repertoire. Data quality should always be considered as a potential explanation for low levels of predictive accuracy from infancy, with need for greater standardisation of quality metrics within particular methodologies. Stochastic developmental processes will also play a role (White, 2019), and these are likely to decrease the strength of association between two variables proportional to their temporal separation. However, this is unlikely to be the only factor that explains moderate predictive validity of early phenotypes. For example, methods like near infrared spectroscopy produce stronger signals in early development than in later development because of factors such as skull thickness (Lloyd‐Fox, Blasi, & Elwell, 2010). Emerging studies indicate that some infant measurements achieve levels of test–retest reliability that are comparable to adult measurements (e.g. brain connectivity,(Haartsen, Jones, Orekhova, Charman, & Johnson, 2019). Further, some sensorimotor predictors of later ASD outcome are often stronger at younger rather than older ages. For example, the speed of identification of a visual target amongst distractors at 9 and 15 months predicts later autism; the same phenotype at 2 years does not (Cheung et al., 2018). Further, the pupillary light reflex relates more clearly to later autism outcome at 9 months than in later development (Nyström et al., 2018). Thus, it is not the case that features that are moderately predictive in early infancy become more strongly predictive later in development. Rather, the decrease in predictive validity of some sensorimotor factors may be indicative of the emerging influence of other systems in the second year of life. Indeed, there have already been empirical demonstrations for significant moderation in developmental trajectories to ASD by sex (Bedford et al., 2016) and effortful control (Bedford et al., 2019). A range of evidence converges to point to the action of protective, resilience or other modifying factors on developmental trajectories to ASD (Chawarska, Macari, Powell, DiNicola, & Shic, 2016; Harrop et al., 2018; Klin et al., 2020; Robinson, Lichtenstein, Anckarsäter, Happé, & Ronald, 2013). Our framework seeks to provide a tractable way to formalise the relationships between these factors.

Despite substantial empirical progress in detecting features that may differ in infants with later ASD, the existing modal analytic approach of looking for group differences in single markers between infants who do or don’t meet criteria for later ASD (or other clinical outcomes) is beginning to limit our ability to break new ground in the study of neurodevelopmental disorders. In the context of our four challenges, this currently popular approach (a) often implicitly (if not explicitly) encourages a focus on single causal pathways because many studies focus on only one or two domains; (b) intrinsically reinforces existing diagnostic categories by beginning with that as the primary unit of analysis; (c) often implicitly adopts one level of explanation through examining one methodology or signal; and (d) through often focusing on single infant time‐points, misses the opportunity to examine complex interactions between risk and resilience. While most studies in this vein explicitly indicate that there are likely many paths to autism, examples of studies in which multiple measures are combined with sufficient power and statistical sophistication to detect this remain rare (though see Constantino, 2018). A range of recent conceptual papers have highlighted important features of an approach that would move beyond an implicit single core deficit framework (Astle & Fletcher‐Watson, 2020; Constantino, 2018; Hong et al., 2020; Klin et al., 2020), including stratifying of ASD ‘outcome’ into more homogenous biotypes (Hong et al., 2020); the need to consider both strengths and weaknesses and to adopt statistical approaches that embrace complexity in trajectories (Astle & Fletcher‐Watson, 2020); and the potential for multiple developmental endophenotypes (some shared with other disorders) to contribute to autism liability (Constantino, 2018). To make significant progress on these ideas, we need to adopt principled theoretical and statistical frameworks through which we can interpret existing data and develop novel study and analytic designs.

In our framework (AMEND), we propose that it is critical to differentiate markers that reflect the activity of brain systems that are highly engaged in sensory and motor functions in early development and primarily implement *early‐stage processing*, and markers that reflect the activity of brain systems that are increasingly influential in modifying later developmental trajectories and could act to promote resilience (specifically, *neurocognitive modifier systems*). In other words, we propose that mappings from perturbations of initial early‐stage processing to later phenotypic outcomes can only be fully understood following the identification of other brain systems capable of modifying the subsequent trajectory of development. This new framework offers a natural position from which to understand existing findings on infants with later neurodevelopmental disorders. Specifically, associations between early‐stage factors and phenotypic outcomes are highly likely to lack individual predictive power due to the missing additional factor of trajectory‐modifying brain systems; early markers may differ in their nature from diagnostic symptoms as the latter are the outcome of a set of complex nonlinear developmental interactions between early‐stage processing markers and neurocognitive modifiers; and phenotypic outcomes in development are the result of multiple interacting brain systems and thus discrete and localisable differences in the brain will not be uniquely associated with outcome in all individuals (Zabihi et al., 2019). Our framework also accommodates insights from other conceptual advances; for example, neurotypes of autism (Hong et al., 2020) may be reflected in different combinations of early‐stage and modifier systems; early‐stage markers may act additively with each other to raise the likelihood of an autism diagnosis (Constantino, 2018); and modifier systems could play an important role in explaining strengths in individual profiles (Astle & Fletcher‐Watson, 2020) and provide fruitful targets for intervention (Klin et al., 2020).

Following a systems biology approach in which it is the interaction between components that determines system‐wide outcomes, we argue that it is now critical to change the current conceptual approach by separating markers of perturbations in the brain systems that underpin early‐stage processing from modifier systems that show more prolonged development. This allows us to embrace a systems neuroscience approach by giving us a framework within which to investigate how genetic and environmental factors shape developmental trajectories through the interaction between higher‐level brain systems. Our framework also provides a natural context in which to study the multiple causal factors that may predispose towards ASD or ADHD and other associated neurodevelopmental conditions that frequently co‐occur with each other, and the modifier systems that may then lead to a canalisation towards attractor states or coherent endpoints; the way in which both categorical and dimensional variation could emerge from the action of modifier systems on earlier‐emerging disruptions to push development towards particular attractor basins; the ability to study mechanisms at the brain system level; and particularly in identifying the modifier systems that could provide protection or promote resilience in the face of earlier risk. Further, understanding neurocognitive modifiers is key for designing effective intervention strategies as they present the best current targets for changing outcome.

## Human neurodevelopment: the case for modifier systems

Like other bodily organs, the brain has multiple homeostatic processes operating at molecular, cellular and systems level. For example, the balance of excitation and inhibition in neural circuits is regulated by homeostatic feedback loops (Nelson & Valakh, 2015). It is likely that similar intrinsic regulatory processes are engaged during development resulting in buffering of particular developmental pathways (‘Chreods’, Siegal & Bergman, 2002). Considering this in terms of developing brain systems in humans, the anterior regions of cerebral cortex, and particularly prefrontal cortex (PFC), have been implicated as being capable of modifying the function and development of other brain systems over multiple time scales. In the following section, we review the evidence that some anterior cortical regions show distinct developmental features that set them apart from posterior sensory cortices.

### The PFC develops for longer than other brain regions

In attributing cause in development, later or more prolonged developmental phases present the opportunity to modify earlier ones. The traditional view of the development of PFC is that it is the slowest developing region of the human brain, and therefore, it was assumed to be the last to ‘mature’. Indeed, in typical human development PFC shows prolonged anatomical development, such as changes in grey matter volume, white matter volume and cortical thickness throughout childhood and into the teenage years (Cafiero, Brauer, Anwander, & Friederici, 2019b; Gogtay et al., 2004). However, while the structural development of PFC continues late into postnatal life, there is increasing evidence that it can be functionally activated and is involved in constructing and regulating behavioural responses during early infancy (Grossmann, 2013). For example, from fMRI resting state analyses and stimulus activation studies the PFC is one of the first active hub regions in the human brain (Fransson, Aden, Blennow, & Lagercrantz, 2011), though some of the resting state networks in which it participates do not clearly emerge until the second year (Gao et al., 2009). These features are distinct from sensory regions of posterior cortex, which show earlier development in both grey and white matter that stabilises around the second year of life (Li et al., 2013). The continuous but highly prolonged trajectory of development of the PFC offers the opportunity for this part of the brain to orchestrate, and potentially modify, the influence of earlier developing regions. This co‐development of regions is likely to be interactive and engage both feed‐forward and feedback pathways, with the PFC potentially having a hierarchical relation to posterior sensory and motor areas (Johnson, 2011).

### Isolation from sensory and motor systems

While the genetic, molecular, cellular, and computational processes that underlie the parcellation of the cerebral cortex into structural and functional areas remains a topic of active research, an important and hitherto neglected factor may be differences in the timing of neurodevelopmental events between large areas of cortical tissue (Cahalane et al., 2011). During prenatal development, projections from the thalamus enter the developing cortex to ‘capture’ their target cortical areas, with one example being the visual inputs into the primary visual cortex from the lateral geniculate nucleus. These sensory or motor connections via the thalamus entrain the still developing cortical neuronal morphology and connectivity, with the result of fine‐tuning the cortical tissue for processing specific kinds of information (Cahalane et al., 2011; Sur & Leamey, 2001). Bearing in mind the importance of these sensory or motor connections it is not surprising that primary sensory thalamic afferents innervate the cortex first to form primary sensory areas. Less obviously, this approximately posterior to anterior innervation by thalamic afferents runs counter to the timetable of development of the cortical neurons themselves, as cortical neurogenesis proceeds in a rostro‐caudal progression with frontal areas being differentiated before more posterior regions (Cahalane et al., 2011). Put simply, there are diametrically opposed gradients of development across the developing cortex that ensures that while the differentiation in some (more posterior) regions is heavily dominated by sensory and motor input, the differentiation of more anterior regions is relatively unconstrained. In these anterior areas (such as the tissue that later becomes PFC) the early morphological development of neurons and their connectivity is more influenced by their intrinsic (spontaneous) activity and patterns of inter‐connectivity with other cortical areas. This intriguing developmental timing produces an anterior portion of the cortex that is comparatively detached from sensory and motor influences and as a result is qualitatively different from posterior sensorimotor regions. Indeed, it has been suggested that PFC may become tuned to orchestrating activity across other regions (Shultz, Rivest, Egri, Thivierge, & Dandurand, 2007). Further, this process may be the developmental roots of the sensorimotor to transmodal cortical gradients that can be identified in the adult brain (Huntenburg, Bazin, & Margulies, 2018) and that may be disrupted in ASD (Hong et al., 2019).

### PFC represents the top level of a hierarchical organisation

During human postnatal development, the brain becomes increasingly hierarchical in its organisation (Supekar et al., 2013). Re‐organisations of whole brain functioning in typical development may occur when new levels of hierarchical control are established, and present opportunities for adaption to minor deviations from the typical intrinsic functioning of regions, or to unusual environments. As part of the basic architecture of the vertebrate brain, the cerebral cortex exerts modulatory control over the more evolutionarily ancient subcortical structures, and some have proposed that PFC stands in a similar relationship to the rest of cortex in primates (Dembrow & Johnston, 2014; Paneri & Gregoriou, 2017). Consistent with this role is the region’s association with ‘executive functions’ (Alvarez & Emory, 2006). As the name implies, this collection of cognitive and behavioural functions (which include inhibition, forward planning and flexibility) are characterised by their controlling influence over other processes. While this association between structure and function is widely accepted, the potential role of PFC in organising other cortical regions during the course of development remains more speculative. Nevertheless, over the past 20 years, this idea has recurred in the literature (Johnson, 2011; Thatcher, 1992), raising the further questions of why PFC is uniquely placed to orchestrate other cortical regions, how it influences these regions, and what role it plays in ontogenetic adaptation.

In the last section, we saw how the neurons that will compose PFC develop with less influence from sensory and motor processing than neurons in posterior regions and are thus likely to more shaped by intrinsic interactions with other cortical areas, a feature entirely consistent with a higher level of organisation. However, this does not explain how PFC orchestrates other brain regions. We have previously (Johnson, 2015) discussed this issue in terms of a type of computer model known as knowledge‐based cascade correlation (KBCC; Shultz et al., 2007). These network architectures can learn many tasks faster, or learn tasks that other networks cannot, because they recruit the ‘knowledge’ and computational ability of other self‐contained networks as and when they are needed. Essentially, a hub network learns to select appropriately from a library of available computational regions to orchestrate the best combination of these for the learning problem at hand. The PFC may act as this ‘hub network’, marshalling the resources of posterior sensorimotor regions where necessary.

### PFC may orchestrate recovery after perinatal brain damage?

Prefrontal cortex may also play a key role in the re‐organisation of cortical functions known to occur following perinatal damage to localised parts of the cerebral cortex. For example, large‐scale prospective studies of language in children who suffered a single focal unilateral injury event to either the right or left hemisphere before 6 months of age show some degree of general developmental delay in measures of language (such as lexical, grammatical and discourse structure) regardless of lesion site (Stiles et al., 2002). However, these delays resolve over time in the children with focal lesions allowing them to score within the typical range. Subsequently, delays can re‐appear at the next steps of development in language acquisition, a pattern consistent with functional recovery to typical performance (resilience) being a reoccurring event during key points of development (Reilly, Bates, & Marchman, 1998). However, outcomes are considerably less positive if perinatal damage is widespread and/or frontal regions are affected consistent with the regions role in neurocognitive resilience (Johnson, 2017). Thus, there is also emerging empirical evidence that the PFC may have a role in organising active compensation for earlier‐emerging differences in posterior brain systems.

### 12 months: A time of transition?

While postnatal brain development in humans is generally a gradual and incremental process that stretches over two decades, there are particular phases in which development may be more rapid, or in which underlying factors may lead to rapid nonlinear change in emergent systems. One such developmental phase is the end of the first‐year postnatal when there are some marked changes in behaviour and brain function associated with a greater degree of PFC control (see Johnson & de Haan, 2015 for review). Improved goal‐driven abilities around this age such as retrieval of hidden objects (object permanence), detour reaching, means‐ends planning, proto‐imperative pointing and deferred imitation have been attributed to development of parts of PFC (such as dorsolateral PFC; for review, see Johnson & de Haan, 2015). More recently, there has been increased interest in this time of transition to greater PFC influence for several reasons. First, and contrary to the prevailing view that the migration of neurons is over by birth in humans, the discovery that there are late migrating inter‐neurons travelling into parts of the prefrontal cortex (Paredes et al., 2016) has raised the real possibility of basic changes in the computations the region can perform, underpinned by changes in the balance of excitation and inhibition. Second, evidence from studies of infants at elevated likelihood of autism has suggested that this period is when behavioural traits characteristic of autism typically emerge. For example, parent concerns become predictive of later autism around 12 months but not before (Ozonoff et al., 2009); reductions in orientation to faces and vocalisation in infants with later autism emerge around 12 months of age on direct observation (Ozonoff et al., 2010); and diagnostic stability increases between 12 and 14–16 months for a proportion of infants who show early symptoms of ASD (Pierce et al., 2019). Thus, transitions to cortical networks biased to PFC around the end of the first year may contribute to the emergence or consolidation of the characteristic features of some neurodevelopmental conditions.

## A new framework: AMEND—Anterior Modifiers in the Emergence of Neurodevelopmental Disorders

Taken together, there is substantial evidence that anterior cortical systems could play a fundamental and increasing role in modifying the effects of lower‐level sensory and motor systems on developmental trajectories. In order to understand the emergence of neurodevelopmental disorders, our new framework is centred on dissociating markers of early‐stage processing from subsequent neurocognitive modifiers. In the sections below, we outline the hallmarks of each type of marker and review existing literature on their role in early ASD.

### Early‐stage processing

Over past decades, nearly every part of the brain has been associated with ASD (Anagnostou & Taylor, 2011; Riddle, Cascio, & Woodward, 2017). Often these studies have been based on small sample sizes and depended on group comparison designs. A more recently emerging view is that there are subtle but widespread anatomical differences across many parts of cerebral cortex (Hazlett et al., 2017; Zabihi et al., 2019). More specifically, converging evidence from genetics and neuroscience indicates the latter stages of the prenatal formation of the human cortex as a developmental time when distal aetiological factors become important. For example, gene expression studies show that the peak expression patterns of ASD‐associated genes are prenatal or very early postnatal and affect a range of different brain regions (Parikshak et al., 2016; Satterstrom et al., 2020; Writing Committee for the Attention‐Deficit/Hyperactivity Disorder et al., 2020). Newly identified outer radial glial (ORG) cells add a level of complexity to the construction of the upper layers of primate cerebral cortex that is not observed in rodent models ((LaMonica, Lui, Hansen, & Kriegstein, 2013; Nowakowski, Pollen, Sandoval‐Espinosa, & Kriegstein, 2016). These non‐neuronal cells provide the support for young neurons to travel from their place of origin to their final destinations in the cortex. Evidence from postmortem tissue and stem‐cell organoids indicate that disruption of ORG leads to discontinuous glial scaffolding (a lack of glial cell guidance for the migration of neurons), differentially affecting development of the upper layer (feed‐forward) pathways of cortex. Converging data from molecular and genetic analyses also implicates ORG and the formation of upper layers of cortex in ASD (Nakagawa et al., 2019), potentially occurring in the late prenatal/early postnatal period in humans. Interacting with, or as a consequence of, these potential distal factors are more proximal factors such as disturbances in excitation/inhibition balance mediated by perturbations to GABA levels (Coghlan et al., 2012), or delayed GABA switching (from excitatory to inhibitory; (Tyzio et al., 2014; Zimmerman & Connors, 2014). Our assumption is that these or similar factors underlie suboptimal processing in sensory and motor parts of cortex, raising the likelihood of later atypical phenotypes such as ASD or ADHD.

The role of atypical sensory processing in ASD has also been recently highlighted through the inclusion of sensory symptoms as part of the diagnostic criteria (DSM‐5; APA, 2013). A substantial majority of individuals with ASD show hyper‐ or hyposensitivity to auditory, tactile or visual stimulation (Robertson & Baron‐Cohen, 2017). Experimental paradigms provide converging evidence of reduced sensory habituation and gating (Green et al., 2019; A. Kolesnik et al., 2019), enhanced visual discrimination (Gliga et al., 2015; Mottron, Dawson, Soulières, Hubert, & Burack, 2006) and altered tactile perception (Puts, Wodka, Tommerdahl, Mostofsky, & Edden, 2014). Brain scanning studies link some of these findings to alterations in sensorimotor networks and their connectivity with other regions (Green, Hernandez, Bookheimer, & Dapretto, 2016). Given the early emergence of sensorimotor function, this raises the possibility that sensorimotor changes could present early indicators of later ASD (Piven, Elison, & Zylka, 2017).

In the AMEND framework, we define markers of early‐stage processing as indicators of the fidelity of sensory and motor processing in posterior cortical areas (Robertson & Baron‐Cohen, 2017), which in turn are assumed to reflect the distal and proximal neural factors that influence synaptic efficiency, excitation/inhibition balance and processing. Such markers of atypicality are also ‘early stage’ in that they are assumed to be present early in postnatal development (and potentially also prenatal), but will have only moderate specificity and sensitivity in relation to atypical phenotypic outcomes due to the influence of later neurocognitive modifiers. Our criteria for markers of early‐stage processing are therefore that: (a) they are observable during the first year and prior to the onset of diagnostic behavioural traits of neurodevelopmental disorders such as ASD or ADHD, (b) they are (ideally replicably) associated at a group level with dimensional or categorical phenotypic outcome, and (c) they reflect atypical sensory or sensory‐motor processing in more posterior cortical systems potentially reflecting differences in the efficiency of synaptic processing and/or excitation–inhibition balance. Of note, we propose that atypicalities in early‐stage markers be initially defined in stage (1) with reference to the normative range (as in the clinic we might identify an infant with an atypically low head circumference relative to WHO norms). This allows for the fact that some atypicalities may be fully compensated in some environments or for some infants, but still be recognised as an atypical starting point for development. Table 1 further elaborates the relation between elements of our framework and the broader concepts of risk, protective factors and resilience in psychiatry.

Early sensory atypicalities have indeed been broadly reported in prospective longitudinal studies of infants with later ASD, particularly in the first year of life. For example, in one longitudinal study, parents of infants with later ASD reported greater behavioural responsivity to perceptual stimuli at 6 months (Clifford, Hudry, Elsabbagh, Charman, & Johnson, 2013); though see (Jones, Dawson, & Webb, 2018). More direct measures of sensory processing have confirmed early alterations. For example, in a longitudinal study involving two cohorts, Nystrom et al showed an exaggerated pupillary response (larger amplitude constriction) to a change in luminance in 8‐month‐old infants with later ASD (Nyström et al., 2018) in the combined sample. Similar findings have been observed in children with an ASD diagnosis (Daluwatte et al., 2013). Also, in 8‐month‐old infants, Gliga and colleagues showed that infants with later ASD were faster to find a target amongst distractors in an eye‐tracking paradigm, consistent with ideas about enhanced perceptual functioning (Gliga et al., 2015). Direct measures of brain activity have also revealed atypicalities. For example, in an analysis inspired by a study of children with Fragile X (Ethridge et al., 2016, 2017), Kolesnik and colleagues (Kolesnik et al., 2019) showed heightened cortical reactivity to auditory tones (characterised by reduced habituation and increased inter‐trial coherence) in 8‐month‐old infants with later ASD. Similarly, Piccardi and colleagues (in press) report reduced tactile gating at 10 months in infants with an older sibling with ASD; similar effects have been noted in children with ASD (Balasco, Provenzano, & Bozzi, 2020). Taken together, these studies point to differences in sensory processing across multiple modalities in infants with a family history of ASD that in many cases resemble the sensory atypicalities observed in older children with a diagnosis.

These sensory atypicalities are also accompanied by early‐emerging motor problems. The presence of a head lag when pulled to sit at 6 months has been associated with later ASD (Flanagan, Landa, Bhat, & Bauman, 2012) and has been incorporated in trial screening programs, along with measures of head circumference (Samango‐Sprouse et al., 2014). Infants with later ASD show subtle alterations in motor skills at their time of emergence (e.g. reaching, grabbing; West, 2019), a profile resembling the multiple waves of resilience following focal perinatal cortical damage described earlier. Other studies have reported atypical postural development in the first year of life, subtle versions of which may be exhibited by siblings who do not go on to develop ASD (Charman et al., 2017). A large longitudinal analysis also confirmed atypical gross motor skills at 6 months in infants with later ASD (Estes et al., 2015). However, a subsequent analysis of a large multisite dataset from the US Baby Sibs Research Consortium indicated that fine motor skills (like grasping, reaching and gripping) at 6 months were related to dimensional variation in levels of ASD symptoms at 36 months, but did not find differences related to categorical ASD outcome (Iverson et al., 2019). Of note, the mixed evidence on specificity to later ASD is consistent with the model that early atypicalities may interact with later neurocognitive modifiers to shape outcome.

Such functional atypicalities are consistent with structural neuroimaging studies of the developing brain in infants with a family history of ASD. In a landmark study, (Hazlett et al., 2017) collected structural brain scans of over 300 infants tested longitudinally from 6 to 24 months. Analysis of surface area indicate increased growth rate between 6 and 12 months in the infants with later ASD, with differences most pronounced in the occipital gyrus, right cuneus and right lingual gyrus areas. A data‐driven approach confirmed that changes in surface area (rather than cortical thickness) were most predictive of later diagnosis. Surface area varies greatly across species and is closely related to functional specialisation (Fish, Dehay, Kennedy, & Huttner, 2008); it also develops in association with white matter myelination in early childhood (Cafiero et al., 2019a). Thus, these structural brain differences are at least conceptually consistent with emerging behavioural and neurocognitive indicators suggesting sensory and motor cortices being amongst those regions associated with likelihood for later developing autism.

### Neurocognitive modifiers

We have argued above for the need to take a systems neuroscience approach in which it is the interaction between components, rather than the individual components themselves, that determine outcome. We have also contrasted early‐stage processing with the more prolonged development of anterior modifier systems supported by PFC. We define *neurocognitive modifier systems* as those brain systems that push individual developmental trajectories towards particular common behavioural phenotypes. Just as the neurotypical developmental trajectory for humans is supported by underlying self‐organising and homeostatic processes, we have argued that certain atypical neurocognitive phenotypes, such as ASD, are the end result of a series of local adaptations (Johnson, 2017; Johnson & Gliga, 2015). In particular, we have marshalled evidence consistent with the view that functions associated with the prefrontal cortex are key for adjusting and regulating more posterior cortical areas over developmental time (Johnson, 2012). Putting together the lines of evidence outlined in Section 2.1, we propose that later developing anterior cortical systems are capable of modifying (compensating or compounding), the activity of earlier developing posterior sensory and motor cortical areas. Behavioural and cognitive markers of these systems will reflect underlying neurocognitive modifier systems.

While we are presenting this framework in relation to early childhood, we note that similar proposals have already been made in the ageing literature. A commonly reported finding from functional neuroimaging studies of healthy cognitive ageing is an age‐related reduction in occipital activity coupled with increased frontal and prefrontal activity. This has led to the hypothesis that the observed posterior–anterior shift in ageing (PASA) is due to prefrontal areas compensating for the decline in function of posterior cortical areas during ageing. For example, in one study (Davis, Dennis, Daselaar, Fleck, & Cabeza, 2008), young and older participants were scanned during episodic retrieval and a visual perceptual task. Age‐related changes in brain activity common to both tasks were identified and revealed results consistent with the PASA pattern even when task performance and confidence levels were matched across the age groups. Further evidence supporting the compensatory hypothesis was that age‐related increases in frontal activity were positively correlated with performance and negatively correlated with the age‐related occipital decreases (but see Morcom & Henson, 2018).

To identify *neurocognitive modifier systems*, we focus on later developing anterior cortical regions and their connectivity, which are key in the active selection of relevant information from the environment during development. Our criteria for identifying neurocognitive modifiers are as follows: (a) they have an increasing influence over neurocognitive development during the first postnatal years; (b) their positive influence is associated with neurotypical developmental outcomes; and (c) they reflect the operation of primarily anterior (frontal) cortical systems that statistically moderates the impact of perturbations to early‐stage processing. Of note, neurocognitive modifiers are designed to be identified at the brain and cognitive level, rather than at the behavioural level at which symptoms of neurodevelopmental disorders are clinically defined. This is important to avoid a circularity between the modelled systems and the disorders in which they are implicated. Although our initial example modifier systems discussed below have been extensively studied in the context of particular diagnostic categories (hence their selection), brain response and neurocognitive measures of those systems are not part of the behaviourally defined diagnostic criteria for these neurodevelopmental conditions per se. Next, we review evidence for two putative neurocognitive modifiers—executive attention and social engagement—and while these are conceptually related to our exemplar conditions ADHD and ASD, respectively, the infant experimental indices by which they are assayed do not have close resemblance to the core behavioural signs and symptoms required for a diagnosis even in preschool children such as overactivity, distractibility and impulsivity (for ADHD) and a lack of reciprocal social smiling, response to name and the presence of repetitive language (for ASD).

One example of a putative neurocognitive modifier is *executive attention* (EA), an anterior cortical system that shapes the inputs to early‐stage processing systems through selecting stimuli from the external environment. EA is evident in a range of top–down strategic influences on looking behaviour. EA can be considered as a component of executive function (EF), but unlike most executive functions can also be assessed in infants (Hendry, Jones, & Charman, 2016). The control and allocation of attention to stimuli is critical as it prioritises the features of the infant’s social and physical environment available for subsequent development and learning. Through regulating interactions with the environment and enhancing or inhibiting the processing of different kinds of stimuli, we anticipate that EA could modify the relation between the activity of posterior cortical areas and later phenotypic outcome. As identified in section 2.1.5, executive attention has increasing influence over behaviour from around 10–12 months postnatal. Strong emerging EA skills could potentially contribute to canalisation through buffering milder disruptions in early brain development like suboptimal signal processing fidelity. If so, this could potentially be a general adaptive response to a variety of different early disruptions.

Executive attention can be measured at behavioural, neurocognitive and brain levels. One of the most replicated early group predictors of later ASD is the speed of oculomotor disengagement in visual attention shifting tasks in which there are competing stimuli. In one of the earliest reports, Zwaigenbaum et al showed slower disengagement from a face to an object at 12 months in infants with later ASD (Zwaigenbaum et al., 2005). This was subsequently replicated in two eye‐tracking studies (Elison et al., 2013; Elsabbagh et al., 2013); Elsabbagh and colleagues further showed that the change in disengagement speed between 7 and 14 months was particularly strongly associated with later ASD (Elsabbagh et al., 2013). While there were no significant differences between outcome groups at 7 months, by 14 months controls and siblings who did not go on to later ASD tended to show a significant reduction in their disengagement latency (potentially reflecting increased executive attention skills); infants who went on to a diagnosis tended not to show this developmental improvement (indicating a lack of emerging EA influence). Further, Elison et al. link atypicalities in disengagement to alterations in white matter tracts potentially reflecting less optimal connectivity between regions (Elison et al., 2013). Extending this work, Sacrey et al showed that, as a group, infants with later ASD tended to be slower to visually disengage from an object during visually guided reaching from 12 months, but not at younger ages (Sacrey, Bryson, & Zwaigenbaum, 2013). One interpretation of these findings is that they reflect a comparative lack of engagement, connectivity or function of EA, and thus, a resulting failure to compensate for atypical early‐stage processing, resulting in a pathway to the ASD phenotype. Contrarily, infant siblings with typical or above average disengagement are less likely to go on to an atypical phenotype. Other measures that tap a similar construct are the duration of longest unbroken look (‘peak look’) during free‐viewing tasks (Hendry et al., 2016). For example, (Hendry et al., 2018) show that infants who showed smaller changes in peak look duration between 7 and 14 months went on to have poorer effortful control at age 3 years, consistent with a link to anterior control systems. Together, the emerging literature could indicate that atypicalities in attentional control are present by the second year of life in infants with later ASD, or that attentional control skills are appearing atypical because they are being used to compensate for other lower‐level motor control issues; use of the AMEND framework can provide a means to disentangle these possibilities.

Atypicalities consistent with attentional control are also observed at the brain level. One way to measure the interrelation between brain systems is to measure neural connectivity, and this has been the focus of a number of infant sib studies (Haartsen et al., 2019; Keehn, Wagner, Tager‐Flusberg, & Nelson, 2013; Orekhova et al., 2014; Righi, Tierney, Tager‐Flusberg, & Nelson, 2014). These studies have commonly examined connectivity while infants watch naturalistic videos, people blowing bubbles, or listen to sounds. Results are mixed; machine‐learning applied to functional connectivity data collected during sleep at 6 months may predict later diagnosis, but the underlying mechanism is unclear (Emerson et al., 2017); 12‐month‐old infants with later ASD may show reduced connectivity between anterior posterior sites in the gamma range during tone processing (Righi et al., 2014) and 12‐month‐old infants with later ASD show decreased intrahemispheric connectivity of oxygenated blood flow (Keehn et al., 2013). Recently, Haartsen et al. (2019) replicated and extended a previous study (Orekhova et al., 2014) and showed that a profile of over‐connectivity in the alpha band between frontal and temporal regions at 14 months was associated with variation in later restricted interests at age 3 years. This is part of the second subdomain of ASD symptoms and has previously been linked to unusually focused attention or ‘monotropism’ (Murray, Lesser, & Lawson, 2005). Because alpha power is often thought to reflect internally generated neural activity (such as cortical ‘idling’, inhibition or suppression) it may be that infants with greater fronto‐temporal connectivity were showing greater internal focus and less responsivity to the social and nonsocial videos on the screen. These atypicalities may reflect a lack of control over attention in order to adjust to different contexts and task demands.

A second potential candidate modifier is the brain system underlying *Social Engagement (SE)*, indexed by markers of attentional engagement to other people and their activities. The social engagement systems we refer to control the attention towards and promote differential processing of visual and auditory scenes with social content. The high heritability of patterns of behavioural social engagement and their disruption in toddlers with autism indicate their fundamental importance in neurotypical development, a key defining feature of a modifier system (Constantino et al., 2017). Critically, social engagement processes are distinct from innate subcortically mediated processes of social orienting (Johnson, Gliga, et al., 2015), and the lower‐level visual and auditory processing of social stimuli (such as that measured by early‐stage responses to faces or voices). Indeed, there is a mounting consensus that early socially specific subcortical orienting mechanisms are intact in ASD (Johnson, 2014). Evidence indicates that progressive reductions in SE over the first two years of life are associated with later autism (Klin, Shultz, & Jones, 2015). Some have proposed that strong SE may be a protective factor (i.e. buffers the neurotypical pathway), particularly in girls with later ASD (Chawarska et al., 2016). It has been suggested that reduced SE may amplify other factors to predispose a child towards ASD (Dawson, Bernier, & Ring, 2012), while increased SE could be protective (Chawarska et al., 2016). Maintaining interest in others despite early‐stage processing differences may buffer children against developing significant social communication problems as they continue to acquire sufficient exposure to the social world.

One important target for future research is to begin to distinguish the influences of social orienting, SE and social information processing on alterations in social functioning in early ASD. In the first year of life, overt social behavioural differences are limited in infants with later ASD (Ozonoff et al., 2010). However, subtler differences have been widely reported. These include a declining trajectory of looking to the eye region of a face between 2 and 6 months (Jones & Klin, 2013), a declining trajectory of face looking from 6 to 24 months (Ozonoff et al., 2010), reduced sensitivity of the temporal lobe to dynamic social stimuli at 5 months (Lloyd‐Fox et al., 2018) and altered neural processing of faces at 6 to 10 months (Elsabbagh et al., 2012; Jones et al., 2016). However, in many cases, the contributory role of lower‐level processing atypicalities has not been elucidated. For example, differences in oculomotor control or in early attention engagement may contribute to difficulties in early visual attention to eyes and faces; indeed, 6‐month‐old infants with later ASD show a profile of shorter fixation durations that could impact social preferences (Wass et al., 2015). Altered cortical responses to motion could also play a role (Nyström, Jones, Darki, Bolte, & Falck‐Ytter, 2020). When neuroimaging datasets comparing face to object processing are examined carefully, atypicalites in object processing are also often seen. For example, Bussu et al. (2020) recently analysed an expanded dataset on neural responses to faces at 8 months (initially reported as (Elsabbagh et al., 2012). Data‐driven analyses confirmed that neural responses to faces were important, but that atypicalities were diffuse and widespread and that neural responses to a nonsocial scrambled stimulus also meaningfully contributed to prediction of later ASD diagnosis. Taken together, the balance of evidence suggests that early sensorimotor atypicalities are not specific to social content but reflect differences in lower‐level processing; however, lower‐level atypicalities do affect social processing (perhaps to a disproportionate degree) from early in infancy.

Novel analytic methods may be required to separate SE from lower‐level aspects of social sensory processing; this may be particularly important in toddlerhood where the two may jointly contribute to behavioural measures of social attention (Constantino et al., 2017). Machine‐learning techniques like microstates can be used to separate hypothetically distinct profiles of cortical signal during event‐related neuroimaging paradigms (Koenig, Kottlow, Stein, & Melie‐García, 2011). Recently, Gui and colleagues used this approach to isolate a microstate with a scalp and temporal profile consistent with measuring attention engagement (Gui, A., Jones, E.J.H. unpublished data). The duration of this microstate was selected as the most informative feature in a genetic‐algorithm model of features predictive of ASD. Further, Bussu et al employed a linked independent component analysis that is designed to identify underlying processes that are manifest in multiple modalities (Groves, Beckmann, Smith, & Woolrich, 2011). Bussu et al. (2020) used this technique to identify modes of covariation in neural responses to faces and infant social behaviour that related to later ASD. Such techniques could provide promising avenues to disentangle the role of different components of social processing at different developmental stages.

## From framework to testable hypotheses

Having established some plausibility for the need to separate markers of early‐stage processing from neurocognitive modifiers, we now consider how to explore the interaction between these factors during development. In particular, we need to move from this general framework to testable hypotheses within the context of prospective longitudinal studies of ASD and ADHD. This requires the selection of appropriate statistical approaches to differentiating early‐stage markers from neurocognitive modifiers and delineating their respective modes of action.

### General analytic strategy

We recommend an analytic strategy that begins with identification of relevant early‐stage processing atypicalities. Candidate measures would be identified by (a) deviation from norm on that measure; and (b) ascertaining general (group level) association with atypical phenotypic outcome. A next step would be to identify candidate neurocognitive modifiers by their association with typical outcomes in those individuals with identified early‐stage processing atypicality. Broadly, neurocognitive modifiers may be initially modelled as *moderators* within a developmental framework. These moderators would be expected to be associated with neurotypical outcomes and thus to act as a transdiagnostic resilence factor. In contrast, early‐stage markers would be most appropriately modelled as predictors. Early‐stage markers would be expected to be associated with known genetic or environmental aetiological risk factors for compromised neurodevelopment. Analytical methods should inform us how the combination of these profiles leads to categorical and dimensional variation in later symptoms.

*Identifying early‐stage markers*. Because alterations in early sensorimotor processing are expected to vary in their relation to later phenotypes based on the action of intervening modifiers, some early‐stage markers could be hard to detect when screening for sensorimotor atypicalities in infants who later meet criteria for ASD. An alternative approach is to obtain evidence for early‐stage markers from infants with known genetic vulnerability. There are a range of well‐known genetic conditions that substantially increase the likelihood of developing ASD or ADHD in later development and that can be identified in infancy (Garg & Green, 2018; Lord et al., 2020). Studying infants with these conditions thus enables the identification of resulting early‐stage sensorimotor atypicalities that could contribute to later neurodevelopmental outcomes (in interaction with later modifiers); these can then be tested in a broader study of both monogenic and familial risk. For example, neurofibromatosis type 1 (Nf1) is caused by a mutation of the gene on chromosome 17 (17q11.2) that encodes neurofibromin, frequently resulting in a later diagnosis of ADHD (up to 50%, Vogel et al., 2017 and/or ASD up to 30%; Garg et al., 2013). About half of the cases of Nf1 are familial, meaning infants can be diagnosed at birth through cordblood testing (Friedman, 1993). The remainder are typically identified in the first year of life, making prospective study tractable. Comparing infants with Nf1 to typically developing infants allows us to robustly identify markers of early‐stage processing that are atypical in the presence of a clear genetic risk factor for neurodevelopmental problems. An initial case‐series studied ten infants with Nf1, identified in infancy and assessed on a broad parent report and observational battery (Kolesnik et al., 2017). Data showed pronounced early motor differences emerging across both parent report and observable behaviour at 10 months, corroborating work in animal models of Nf1 that show disruptions to early‐stage sensory processing (Lush, Li, Kwon, Chen, & Parada, 2008). Other research groups have initiated research studies of infants with tuberous sclerosis (Dickinson, Varcin, Sahin, Nelson, & Jeste, 2019) or deletions on chromosome 22q (Chawner et al., 2017), holding promise for taking a cross‐disorder approach to early‐stage marker identification.

An alternative that could be of utility in a general population sample would be to use techniques like normative modelling (Marquand, Rezek, Buitelaar, & Beckmann, 2016) to identify infants whose brain function measures lie more than a number of standard deviations away from the population mean. This would be akin to defining low birth weight infants relative to population‐specific norms. This method allows identification of infants with atypical early profiles independent of later phenotypic outcome. Critical here is to take into account measurement precision, because atypical values may be more likely to reflect the influence of noisy measurement. Precision can be calculated through metrics like split‐half or test–retest reliability and can be used to refine measurement parameters (e.g. Haartsen, van der Velde, Jones, Johnson, & Kemner, 2020); these should be examined across the full range of measurements. Further, precision can be significantly improved by the use of repeat testing.

### Moderation and additive, multiplicative or interactional effects

Identifying moderation is more challenging than many appreciate. A simple test for statistical interaction of early‐stage marker and later‐stage moderator M on outcome is almost never sufficient. In the case of a binary moderator, even the direction of the interaction can change with the scale on which effects are measured, or correspondingly, are combined. One exception is the instance of qualitative moderation, where the early risk is increased in one moderator group and decreased in the other. Whether effects combine additively or multiplicatively (crudely conceptually equivalent to the outcome scale being log transformed or logit when the outcome is binary and rare) can change the significance and even the direction of the interaction term (Blot & Day, 1979). We have previously illustrated this in the case of early markers for autism (Bedford et al., 2014). For binary outcomes, it is generally agreed that the additive scale (which focuses on the absolute levels and differences in the rate of the outcome) is appropriate for public health purposes. In contrast, in our framework, the focus is on mechanism and the scientific model implies sequential stages, with the early risk factors acting at the first stage to generate a vulnerable subpopulation upon which later moderators act. In this case, the multiplicative model is appropriate (Pickles, 1993)—with the understanding that the absence of an interaction does not necessarily imply a lack of moderation of public health relevance. This multiplicative combining of effects applies whether the intermediate stage is observable or not. However, the above is complicated by the inconsistent impact of different sampling designs on the validity of different analysis models. This means that multiplicative and additive models may be particularly fruitful in the context of prospective general population studies (and to an extent, prospective sibling studies), while their scope for application to case–control (retrospective) designs is more limited.

### Moderators and confounding

The importance of control for confounding for main effects is well understood. However, for interactions, we need to consider not only the confounders for the early risk marker, but also those of the moderator. In a worked example, (Pickles et al., 2013) studied whether social environment (as measured by maternal sensitivity) moderated the effects of the MAOA gene on child anger‐proneness. Analyses considered additional interaction effects of both the moderator and the early risk marker with a range of potential confounders that included child gender, deprivation, maternal age, education, marital status and negative temperament. Of these several were additionally significant, indicating that the interaction may reflect the moderation by a wider construct than maternal sensitivity specifically and potentially the scope for the construction of an interaction effect index that combined several moderators (Cai, 2010). That study also highlighted that the MAOA allele that was responsive to maternal sensitivity differed between boys and girls. Thus, detailed consideration of confounders can substantially alter interpretation even when the estimate of target interaction itself—here MAOA x maternal sensitivity—remains apparently unchanged and significant. While more thorough than most, that study was unable to properly account for the potential confounding of maternal genotype, whose effects may have been mediated by maternal sensitivity. It should also be noted that measurement error, with the possibility that we are measuring an indicator of the moderator rather than the moderator itself, can not only reduce our power to detect moderation but can mean that the target interaction effect is misattributed to the interaction with a better measured confounder. Instrumental variable analysis, essentially a tool to extract from the variation of a variable that part that conforms to a natural experiment, is now a common method of analysis to tackle these problems in epidemiology (Hernán & Robins, 2006) and could be a useful technique to bring more broadly into developmental studies with large samples..

### Multivariate growth curve and trajectories

The analyses above can be considered as being constructed from a prospective perspective, identifying potential causal factors and examining their impact on later development. A somewhat different approach, particularly helpful where we are examining development over a profile of measures simultaneously, is to use a (preferably model‐based) data‐reduction tool such as multivariate growth or trajectory model to reduce variation to a limited number of distinct parameters (such as random intercepts and slopes) or groups (latent classes). These can then be examined for their association with early risk markers and moderators (Hendry et al., 2020). While such an approach can give considerable insight, the feature of the first stage in which information from multiple time‐points is exploited simultaneously can make establishing the causal status of the associations with moderators operating within the interval of those time‐points difficult.

For the purposes of illustration, we imagine a project that involves the prospective longitudinal study of infants with a family history of ASD and/or ADHD alongside controls. In accord with the framework outlined above, we investigate whether candidate neurocognitive modifiers moderate the mapping between early‐stage processing disruptions and later phenotypic outcome. For example, testing that stronger executive attention will be broadly protective from atypical outcome (Johnson, 2012), such that strong EA will be associated with a weaker relation between early‐stage processing markers and both ASD and ADHD categorical and dimensional outcomes. Further, we consider evidence that strong SE will alter the relation between early‐stage processing and later ASD but not ADHD outcomes. In this way, modifiers may confer outcome specificity from the same profile of early‐stage processing differences.

**Figure 1:**
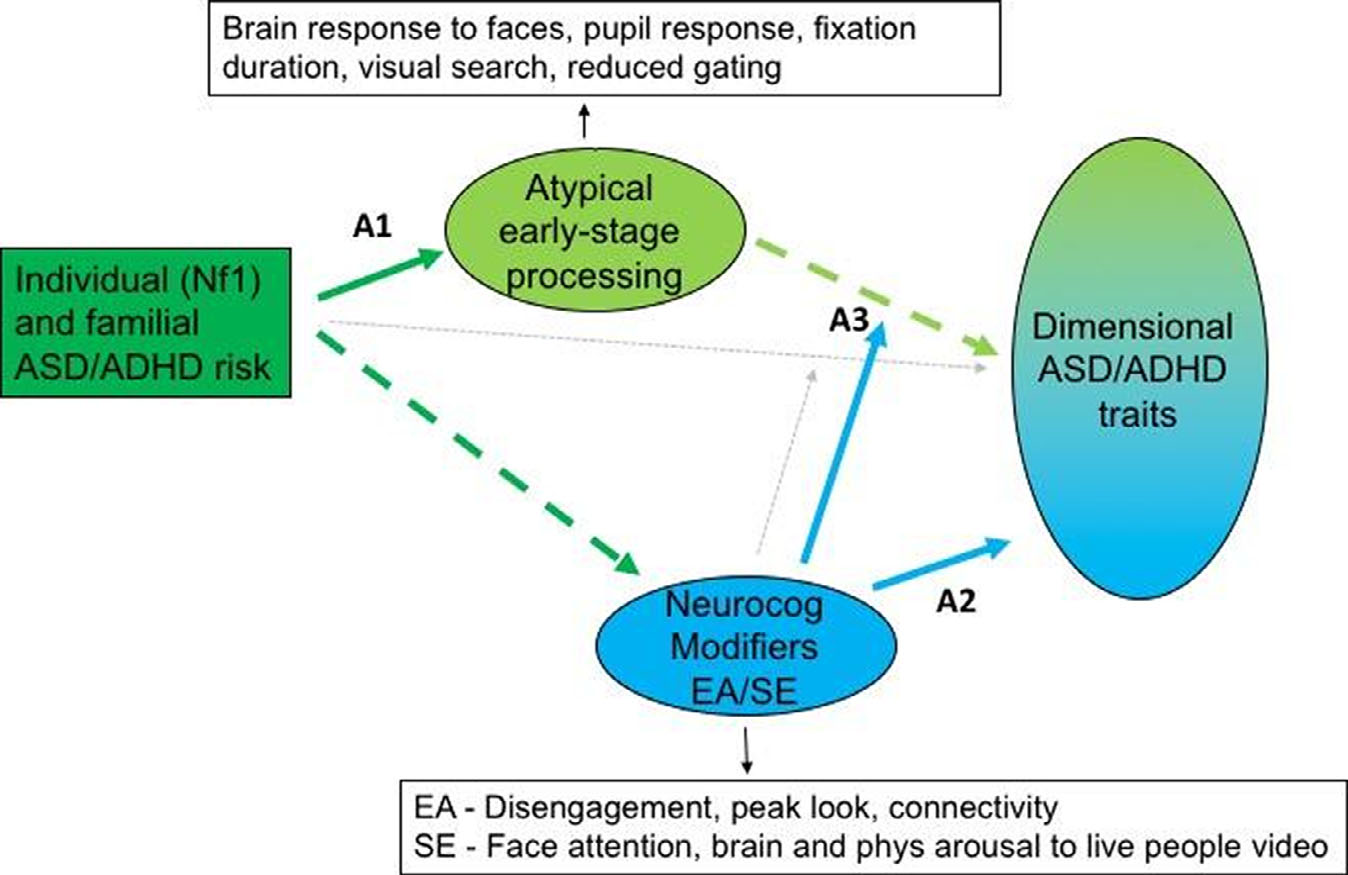
Illustration of the analysis steps motivated by the AMEND framework. Arrows A1 to A3 are described in the text. Colour green indicates the influence of initial factors and Early Stage Processing, while blue indicates the influence of Neurocognitive modifiers. Phenotypic outcomes are represented on the right hand side as resulting from a combination of these factors

Figure 1 illustrates our framework and how it could be used to test specific hypotheses. A1 in the figure illustrates that we can test the hypothesis that specific early‐stage processing atypicalities are present in infants with familial or individual risk for ASD/ADHD. To do this, we could compare the profile of markers of early‐stage processing in infants with an identified genetic disorder such as NF1 versus controls and then compare this with infants with a family history of ASD and/or ADHD, all within the same multigroup structural equation modelling framework (SEM).

A second set of hypotheses (indicated as A2 in the figure) addresses whether certain anterior neurocognitive systems (such as those subserving social engagement and executive attention) promote typical developmental outcomes. This could be tested by using regression and trajectory modelling to examine the relation between markers of these systems and typical development of social adaptive skill and executive functioning within a discovery sample of typically developing controls and where indicated then within the high‐likelihood cohorts. Finally, A3 in the figure assesses whether these anterior neurocognitive systems moderate the effects of early‐stage processing atypicality on the later emergence of ASD and ADHD traits. For A3, all high‐likelihood cohorts would contribute to testing moderation of the shared/common pathway mediated by early‐stage processing atypicality. While the bold arrows in the figure indicate primary hypotheses, we recognise that there may be residual direct effects (dashed arrows) that could also be tested with the cohorts contributing depending upon the risk path. Again, SEM provides a natural analysis framework.

**Figure 2:**
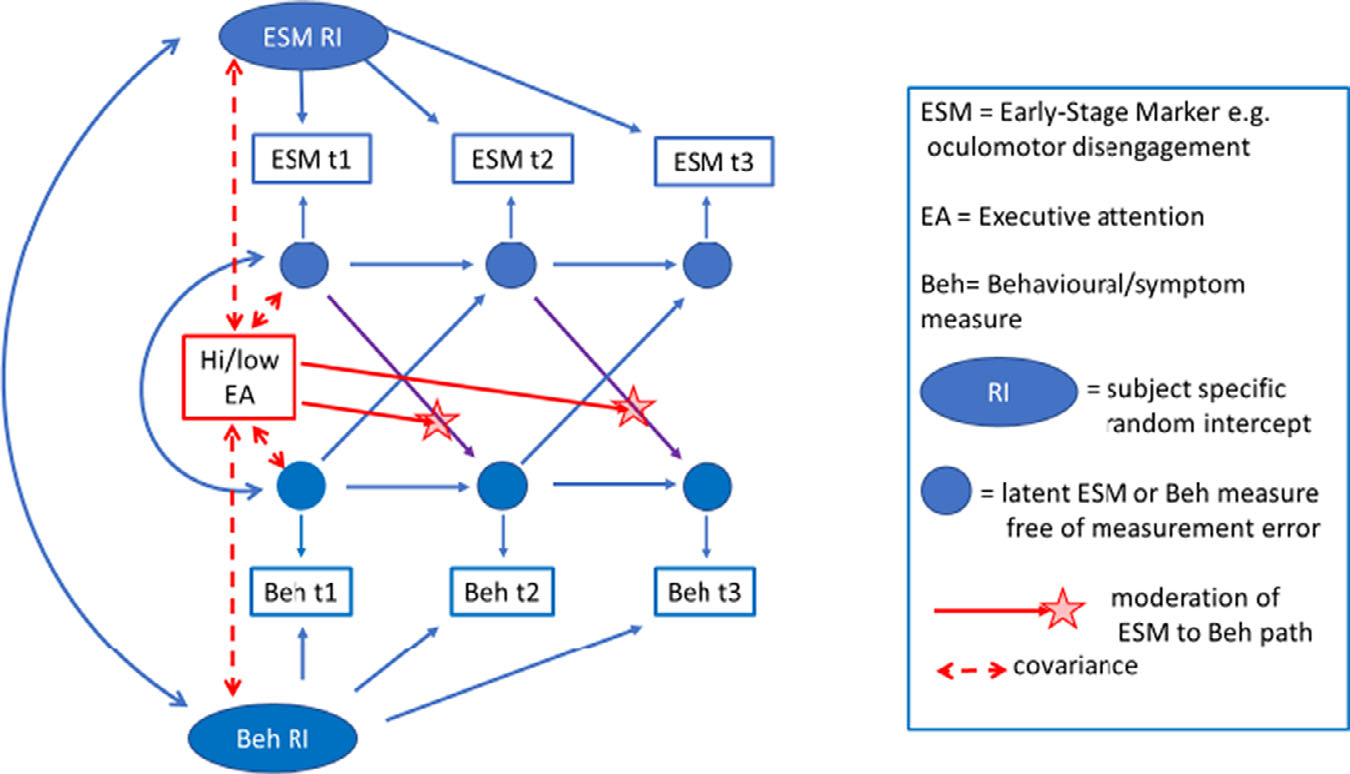
Illustration of a random‐intercept cross‐lagged model to examine how executive attention might over time (through the red paths), reduce the magnitude of (purple) paths that reflect the cumulative impact of an early‐stage marker on later phenotypic outcome

The possibility of unmeasured confounders and reciprocal effects between variables further complicate analysis for which dynamic panel data models have been proposed. One such is the Arellano–Bond model (Arellano & Bond, 1991) which constructs a complex set of instrumental variables to provide an estimator of the primary cross paths of interest that can claim greater formal legitimacy. (Hamaker, Kuiper, & Grasman, 2015) argue that the causal validity of the estimates from the commonly applied SEM with paths between observed variables can be improved by the inclusion of a time‐constant shared random intercept and decomposing each variable into a true‐score factor and a measurement error to account for unreliable measurement. Similar to that used by Carter‐Leno et al. (Carter Leno V, Wright N, Pickles A, Bedford R, Zaidman‐Zait A, Kerns C … & Elsabbagh, M., Unpublished data) who examined EF moderating the impact of life‐events, Figure 2 illustrates a random‐intercept cross‐lagged model (RI‐CLM) of the form that might be applied to examine how executive attention might over time (through the red paths), reduce the magnitude of (purple) paths that reflect the cumulative impact of an early‐stage marker on the development of symptomatic behaviour.

### Maximising power

Can we avoid the proposed framework of interaction dissipating statistical power with the consequent risk of false‐positive findings? Careful variable selection is essential to reduce multiple testing. One key factor is that markers of early‐stage processing are defined with respect to normality (e.g. as extremes within a distribution of a particular brain measurement, or as features seen in infants with a known genetic disorder). Power to detect moderation of their effects depends markedly on the prevalence and correlation between early‐stage marker and modifier, and the form of the moderation. In standard cohort designs, low moderator prevalence or a high correlation typically result in marked imbalance in the numbers in informative cells (or quadrants of the distribution for continuous measures) and thus lower power. Where moderator changes the direction of effect or where there is evidence for no effect of modifier status amongst the low‐risk group sufficient to justify the omission of the main effect of the modifier, then power is often good. Thus, as where evolutionary theory suggests opposing effects or effects in one sex only (e.g. Aiken & Ozanne, 2013; Braithwaite, Pickles, Wright, Sharp, & Hill, 2020) and the vulnerability and provoking agent model of (Brown & Harris, 1986) where the provoker effects only the vulnerable, circumstances where the anterior modifier has no effect on those with typical early‐stage processing can be exploited to advantage.

## Implications of the new framework

In the AMEND framework, we have argued for the dissociation between markers of early‐stage processing that reflect disruptions to perinatal brain function, and neurocognitive modifiers that affect the translation to later phenotypic outcomes in the presence of early‐stage developmental perturbations. We have then suggested ways in which the inter‐relations between early‐stage processing and neurocognitive modifiers could be analysed to test specific hypotheses about these components and their interaction. We believe that this new framework has significant implications for a number of clinical, translational and basic science issues.

First, we note that current polygenic scores are typically constructed through identifying genetic variation that associates with currently defined clinical categories. This approach will inherently mix together genes that might primarily disrupt early‐stage sensorimotor systems, and those that might disrupt later‐emerging neurocognitive modifiers. This is also true of rare variants that are more penetrant and raise the likelihood of ASD and other associated conditions (commonly also intellectual disability). Indeed, analysis of profiles of gene expression indicate that there are different identifiable clusters of genes associated with neurodevelopmental disorders that have different peak expression windows, although whether timing of gene expression maps onto timing of phenotypic expression of a particular trait remains unclear (Satterstrom et al., 2020). An important future endeavour is to create refined polygenic scores that are specific to particular components of the developmental pathway and might explain significantly more variance in end state phenotypes. Indeed, consistent with our framework are recent attempts to derive Polygenic Resilience Scores; designed to detect heritable variation that promotes resilience by reducing the penetrance of risk loci (Schizophrenia Working Group of the Psychiatric Genomics Consortium, & Lundbeck Foundation Initiative for Integrative Psychiatric Research (iPSYCH), 2019).

Second, emerging results from prospective longitudinal studies of infants with a family history of ASD and/or ADHD have re‐emphasised the complexity of the mapping from initial brain function to later outcome in developmental psychopathology. On the one hand, multiple developmental pathways can lead to the same phenotypic outcome (‘equifinality’), while on the other different outcomes can share common early factors (e.g. ASD and ADHD; ‘multifinality’, (Cicchetti & Rogosch, 1996)). Our new framework offers the potential for greater understanding of these phenomena. For example, some components of atypical early‐stage processing could be common to both ASD and ADHD, with different neurocognitive modifiers generating different phenotypic outcomes. Alternatively, different early‐stage processing profiles could be channelled into a common outcome through the action of neurocognitive modifiers (Johnson, Jones, et al., 2015).

We finish the review by proposing that behavioural and cognitive interventions may be most effectively targeted at neurocognitive modifiers. This approach allows one to both test potential interventions in a developmentally and empirically coherent manner and at the same time provides additional empirical evidence itself for the ‘modifier status’ of putative modifiers to stand alongside the prospective statistical modelling approaches outlined above. The convergence of evidence from naturalistic longitudinal studies and mechanistically motivated intervention trials has a long history in developmental psychology (Bradley & Bryant, 1983) and more recently in the developmental psychiatry field (Green & Dunn, 2008). Recent trials have begun to target the age points and brain systems associated with potential neurocognitive modifiers. One prodromal intervention in young infants with an older sibling with ASD used a parent‐mediated intervention modified from the Video Interaction for Promoting Positive Parenting (VIPP) that aims to increase parental attunement and sensitive responding to their infants social communication and thus enhance social attention and social engagement (iBASIS‐VIPP; Green et al., 2017; Juffer, Bakermans‐Kranenburg, & van Ijzendoorn, 2012). After the 4‐month intervention period and follow‐up to 36 months of age, there were reductions in observational measures of early ASD traits and also in parent attentiveness/sensitivity and child attention/communication initiation. Another trial in infants with older siblings with ASD found improvements in neurocognitive measures of social attention and social processing following a similar parent‐mediated intervention (Jones, Dawson, Kelly, Estes, & Webb, 2017). However, a trial with infants age 12 months with early behavioural signs of ASD did not find at endpoint changes on ASD or dyadic parent or child interaction measures (Whitehouse et al., 2019).

In places we have highlighted how hard it is to make an attribution of a causal effect to a moderator with the confidence required to argue that the moderator should be a target variable for treatment, say a parent training, where reducing/increasing the moderator would result in an overall reduction/increment in the outcome. However, in some cases, the purpose is to identify a subgroup, perhaps to receive some treatment whose therapeutic value has been shown by quite different studies. In this case, provided the pattern of the confounders does not vary between study and target population, then it makes no difference whether the effect of the moderator is explained by confounders nor that the moderator might be an indicator rather than the actual causal variable. VanderWeele & Knol (2014) describe a number of other circumstances where analyses, while imperfect from the point of view of causal mechanism, can nonetheless recover estimates of quantities of interest particularly where it can be assumed that the early risk can be assumed assigned at random.

## Conclusion

In this review, we propose the AMEND framework as a tool to reframe the field of prospective studies of neurodevelopmental disorders. Specifically, we propose conceptual, statistical and methodological approaches to separating markers of early‐stage processing from later developmental modifiers. This represents a shift towards a systems‐biology approach to understanding the emergence of typical and atypical neurodevelopment. Separating genetic and environmental factors associated with early‐stage processing and modifier systems may elucidate the aetiology of the shared vs distinct aetiological factors for co‐occurring neurodevelopmental conditions like ASD and ADHD. Finally, studying how different profiles of early risk and later resilience interact to produce neurodevelopmental outcomes may illuminate the categorical or dimensional nature of developmental psychopathology itself. Clinically, differentiating modifiers from early atypicalities may indicate optimal routes for intervention because of their later developmental action and their capacity to alter trajectories and optimise outcomes. Taken together, this approach could significantly advance our theoretical understanding and clinical approach to the emergence of developmental psychopathology in early childhood.
